# Supplementary material for: Polycyclic aromatic hydrocarbon formation chemistry in a plasma jet revealed by IR-UV action spectroscopy
Source: Nat Commun. 2020 Jan 14;11:269. doi: 10.1038/s41467-019-14092-3 (PMC6959308; doi:10.1038/s41467-019-14092-3)
Supplement: Supplementary file 1 — Supplementary Information [file 41467_2019_14092_MOESM1_ESM.pdf]

Supplementary Information

PAH formation chemistry in a plasma jet revealed by IR-UV action spectroscopy

Alexander K. Lemmens et al.

Supplementary Figures

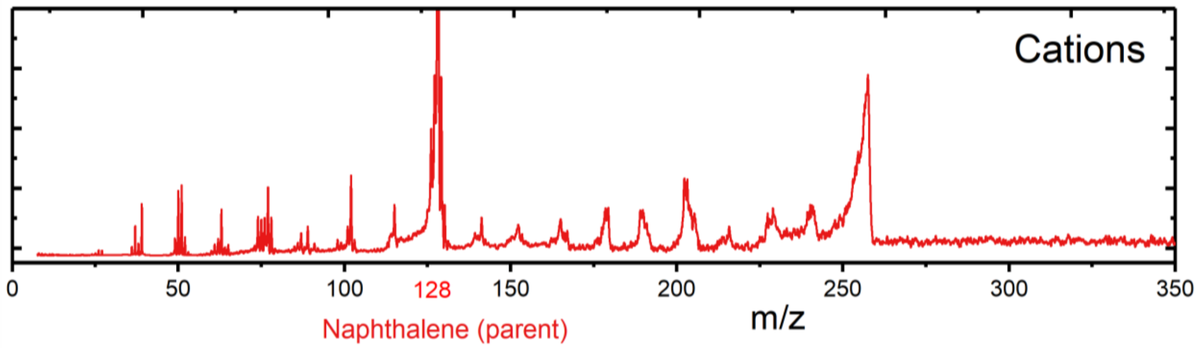

__Supplementary Figure 1: Mass spectrum of cationic products and fragments from naphthalene formed in electrical discharge.__ The cations are injected in the time-of-flight mass spectrometer by applying a pulsed potential to the repellor and extractor plates. Although also dependent on detection settings of the TOF, the cationic mass spectrum gives some indication on the relative abundance of the created products and fragments; however, species with a large mass difference should not be directly compared.

Supplementary Discussion

In this section of the Supplementary, a full overview is given of all relevant calculated IR spectra of probable structures that are compared to experimental IR spectra. As important as assignment, also exclusion of intermediates and reaction products is illustrated in the Figures SI.2-6. The calculations of the structures that are assigned are performed using anharmonic theory, while the calculations of rejected compounds are on the harmonic level of theory.

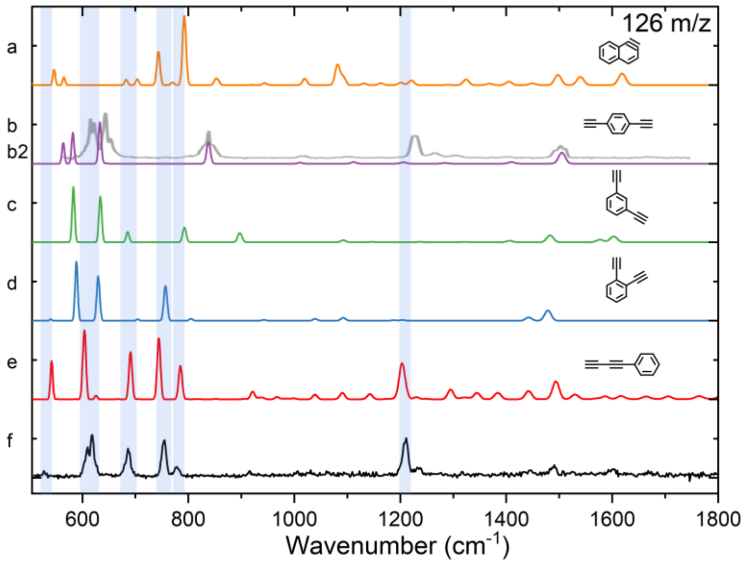

__Supplementary Figure 2: Comparison of calculated infrared spectra of possible isomers for the 126 m/z channel to the experimental IR spectrum.__ The calculated spectrum of the isomer buta-1,3-diyn-1-ylbenzene (diacetylenebenzene, red trace, e) is assigned to experimental spectrum (black, f). The light-blue shaded bands show the best match to the spectrum buta-1,3-diyn-1-ylbenzene, i.e. all bands in the shaded area are present in both the experimental as the theoretical spectrum, which is not the case for the other theoretically calculated spectra (traces a-d). Especially the anharmonic 1210 cm^{-1} band that is present in the experiment (and absent in harmonic calculations) is indicative of a ≡CH group.[1–5] Importantly, based on this comparison, species with mono-acetylene side groups can be excluded (b-d). The gray trace is an FTIR spectrum taken from [2] of 1,4-diethynylbenzene, that is recorded to illustrate the match between theory (purple, b2) and experiment (gray, b). The 1210 cm^{-1} band in the gray experimental FTIR spectrum is again the result of the ≡CH group.[1,2]

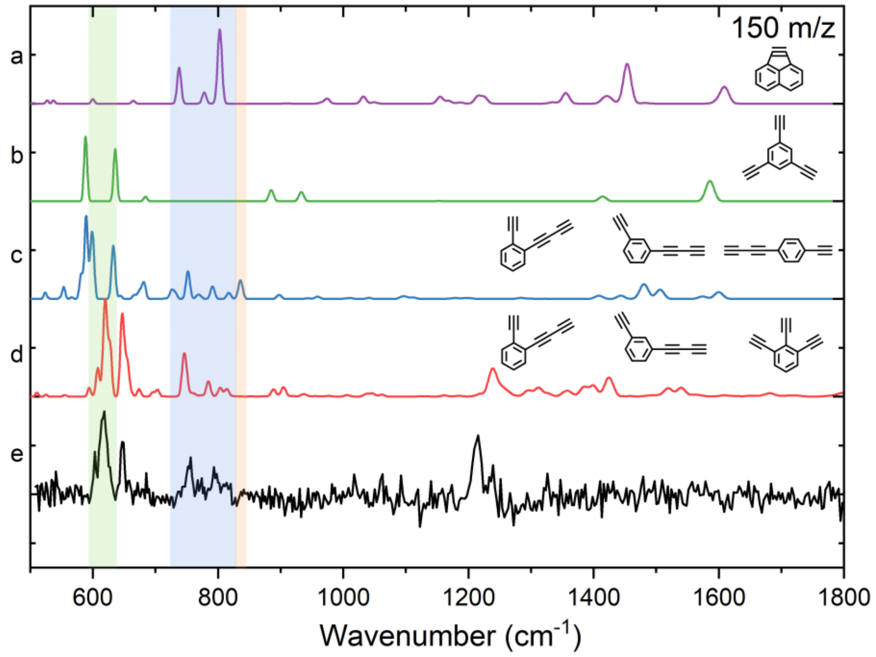

Supplementary Figure 3: Comparison of calculated infrared spectra of possible isomers for the 150 m/z channel to the experimental IR spectrum. *The calculated spectrum composed of the three isomers 1-(buta-1,3-diyn-1-yl)-2-ethynylbenzene, 1-(buta-1,3-diyn-1-yl)-3-ethynylbenzene and 1,2,3-triethynylbenzene (red trace, d) is assigned to experimental spectrum (black, e). The theoretical spectrum a (purple) is not assigned to the experiment based on the absence of IR intensity in the green shaded region. The theoretical spectrum b (green) is discarded based on the absence of intensity in the blue shaded region. The assignment of theoretical spectrum d (red) over c (blue) is a result of the better predicted relative intensity of the green shaded doublet peak as well as the absence of a peak in the experimental spectrum in the orange shaded region. The 1210 cm^{-1} band that is present in the experiment is indicative of a ≡CH group.*[1,2]

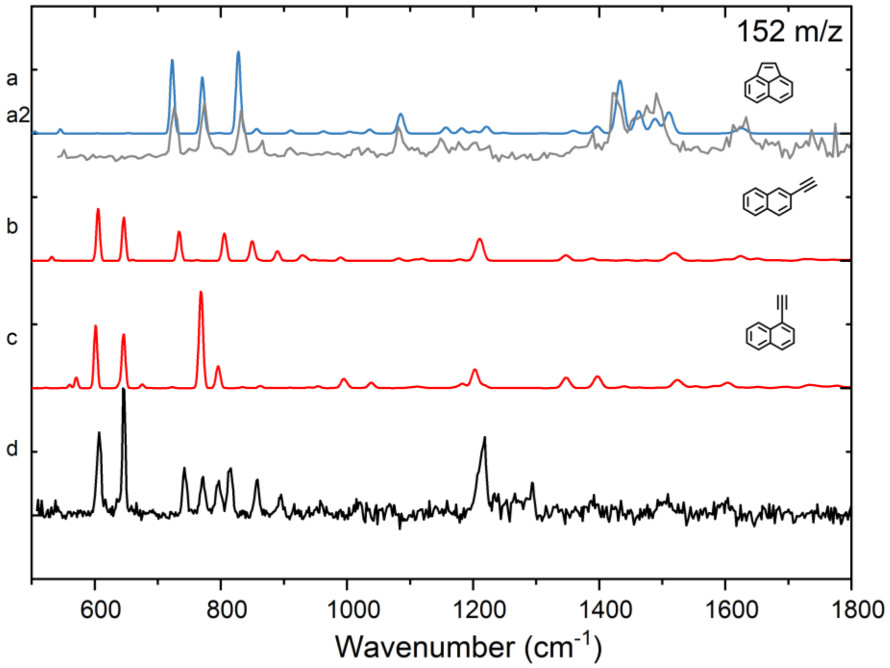

Supplementary Figure 4: Comparison of calculated infrared spectra of possible isomers for the 152 m/z channel to the experimental IR spectrum. *A combination of the calculated spectra of 1- and 2-ethynylnaphthalene (red, b and c) replicates the black experimental spectrum. Importantly, the frequently proposed acenaphthylene (blue, a) is excluded as a reaction product as a result of the absence of infrared bands in the 1400-1600 cm^{-1} region as well as the fact that the experimental spectrum is fully described by a combination of the of 1- and 2-ethynylnaphthalene spectra (see Supplementary figure 7). The gray trace is a jet cooled IR ion dip spectrum of acenaphthylene recorded using the method described in [6], that is recorded to illustrate the match between theory (blue, a) and experiment (gray, a2).*

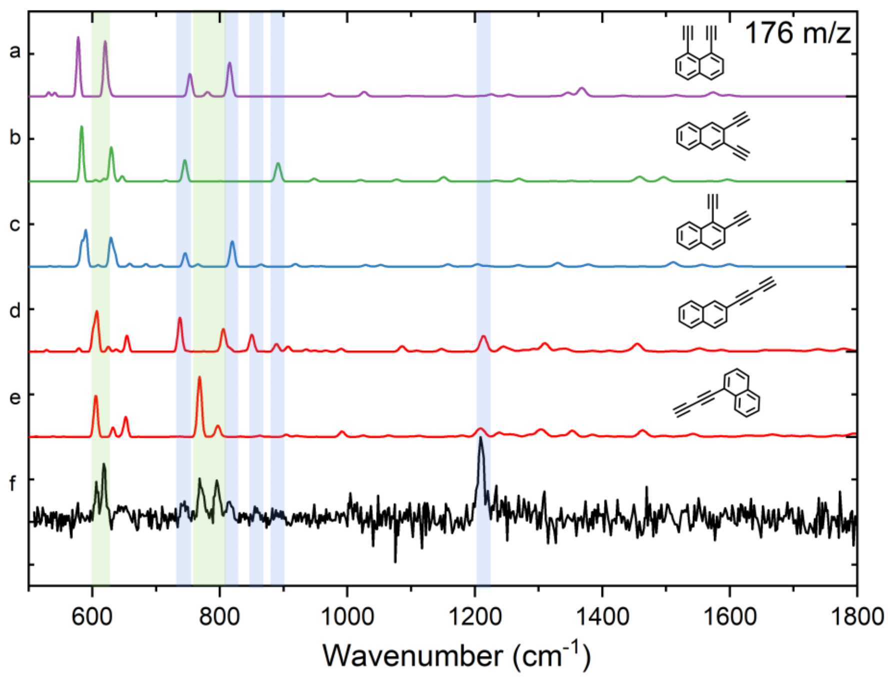

Supplementary Figure 5: Comparison of calculated infrared spectra of possible isomers for the 176 m/z channel to the experimental IR spectrum. *The calculated spectra of both 1- and 2-(buta-1,3-diyn-1-yl)naphthalene (red, d and e respectively) are assigned to experimental spectrum (black, f). As was the case for the above examples, the species with mono-acetylene side groups are excluded based on this comparison (traces a-c). Namely, a doublet with the correct spacing is observed only for species with di-acetylene side groups. In addition, the two bands in the green shaded region are only predicted in the theoretical spectrum of 2-(buta-1,3-diyn-1-yl)naphthalene (e) and the pattern marked by the blue shaded area is described fully by the 1-(buta-1,3-diyn-1-yl)naphthalene theoretical spectrum (d). The 1210 cm^{-1} band that is present in the experiment is indicative of a ≡CH group.*[1,2]

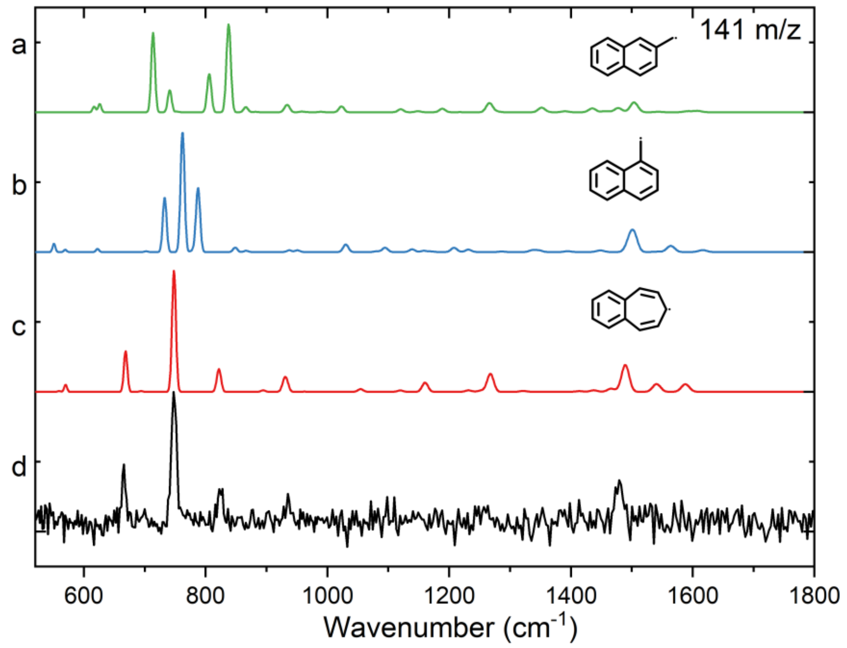

Supplementary Figure 6: Comparison of calculated infrared spectra of possible isomers for the 141 m/z channel to the experimental IR spectrum. *The calculated spectra of benzo[7]annulene (red, c) is assigned to experimental spectrum (black, d). Methyl-radical compounds (a-b) are excluded based on this comparison and the benzo[7]annulene isomer is assigned as the IR pattern is fully described by its theoretical spectrum.*

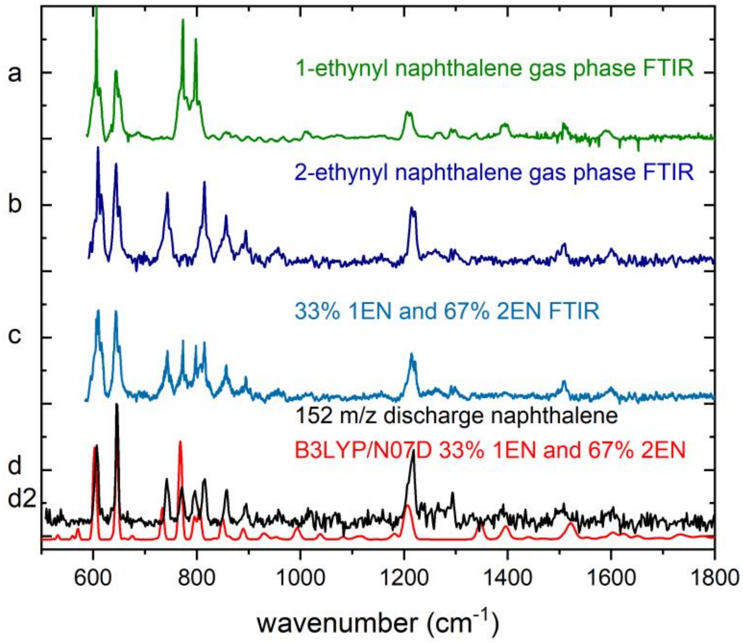

Supplementary Figure 7: Comparison of reference compound FTIR gas phase measurements compared to both experimental discharge (d, black) and calculated spectra of the two ethynyl-naphthalene isomers (d2, red). *Excellent agreement is achieved between FTIR and discharge spectra using a 2:1 ratio of 2EN and 1EN respectively (b and c respectively), including the anharmonic band at 1210 cm^{-1}. This anharmonic band is present in the anharmonic calculations (red, d2). The 2EN FTIR spectrum is by courtesy of P. Constantinidis.*

Supplementary Methods

In order to achieve sufficient cooling in our molecular beam, the widely used linear discharge nozzle (Supplementary Figure 1a) has been adapted. The geometry in Supplementary Figure 1b contains the new design discharge nozzle used in our experiments: a compression zone after discharge but before expansion (Supersonic Compression Outstanding OuTflow Electrical Reactor: SCOOTER nozzle). The new design ensures better rovibrational cooling of the molecular beam (see Supplementary Figures 2-3). Geometry a is used to show that not having compression after discharge results in less efficient cooling (linear nozzle).

The compression after discharge results in a better expansion into the vacuum chamber (Supplementary Figure 2, bottom) with respect to no compression after discharge (Supplementary Figure 2, top) illustrated by computational fluid dynamics simulations (Autodesk Inventor). The compression enables a larger pressure difference upon expansion.

Figure 3 shows 3 different nozzle types (2 linear, 1 SCOOTER nozzle) with the resulting REMPI spectra with both the high voltage applied (red) and without the high voltage applied (orange). They are compared to the REMPI spectrum obtained using only our General Valve. The two broad features correspond to leakage of dissociation of dimer leaking into the monomer mass channel. Upon addition of any discharge nozzle, the REMPI is broadened (corresponding to less efficient cooling), where in the case of the 3 mm linear nozzle, the effect is worst. In case the discharge is turned on, only the SCOOTER nozzle shows resolvable bands at the position where originally two sharp peaks are present. This shows the SCOOTER nozzle ensures better rovibrational cooling and is therefore beneficial to our IR-UV ion dip experiments.

Optimal conditions concerning the discharge are determined from the current vs. m/z 3D plot as shown in Supplementary Figure 4. The scalebar indicates the ion signal intensity. At too high current, all products with larger mass than naphthalene are fragmented and end up in low m/z channels. A optimal current of around 50 mA was determined.

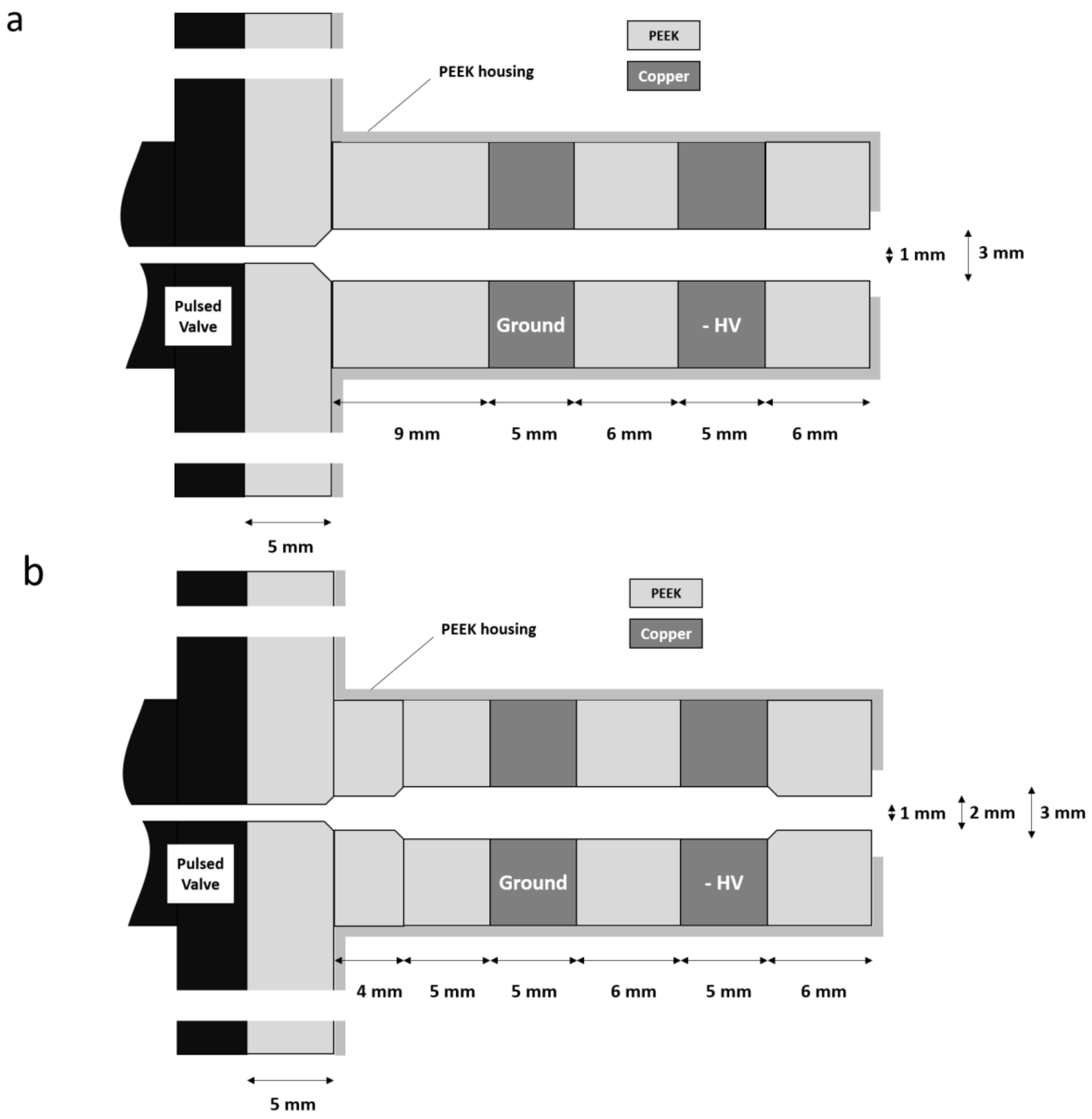

Supplementary Figure 8: Geometry of two different discharge nozzles.

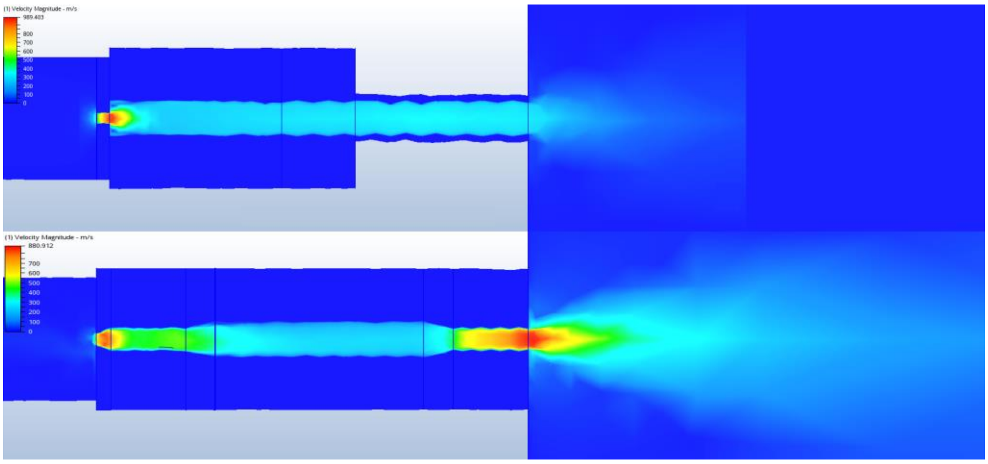

Supplementary Figure 9: Computational fluid dynamics performed on both nozzle geometries.

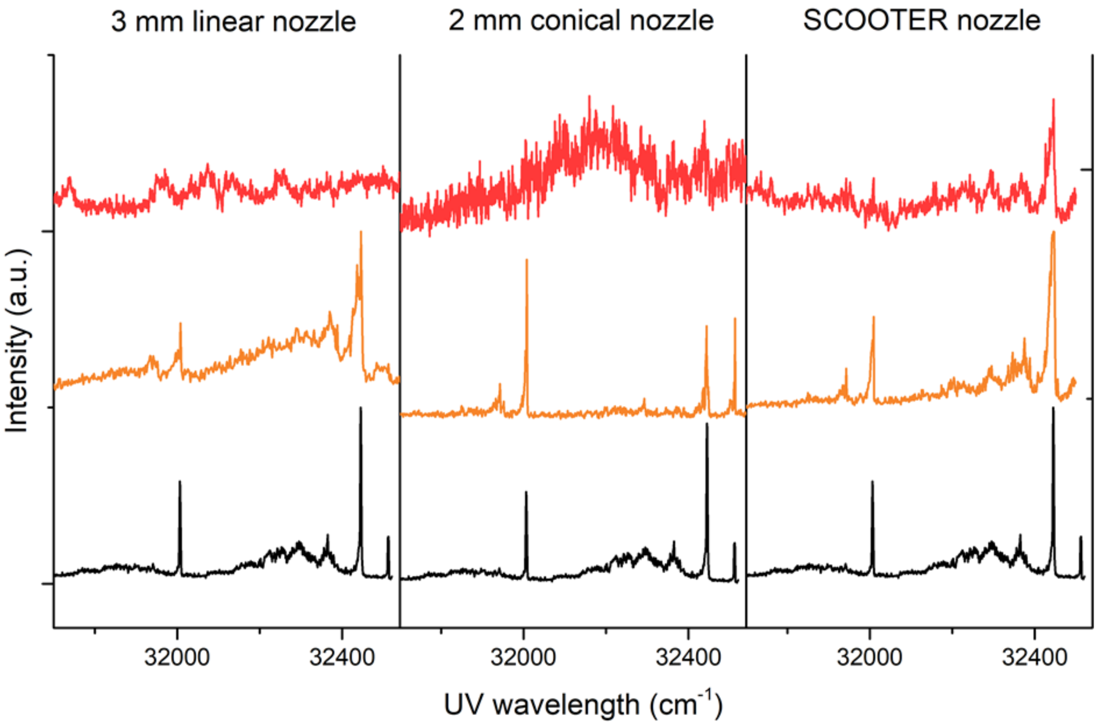

Supplementary Figure 10: UV spectra of naphthalene showing approximate temperature of molecular beam for three nozzle geometries. *The black trace is made without nozzle, the orange trace with nozzle but no discharge, the red trace is with running discharge nozzle. The SCOOTER nozzle yields the lowest rovibrational temperature of our molecular beam after discharge compared to the 2 and 3 mm linear nozzles. 2.5 bar He backing pressure is used for all three REMPI spectra.*

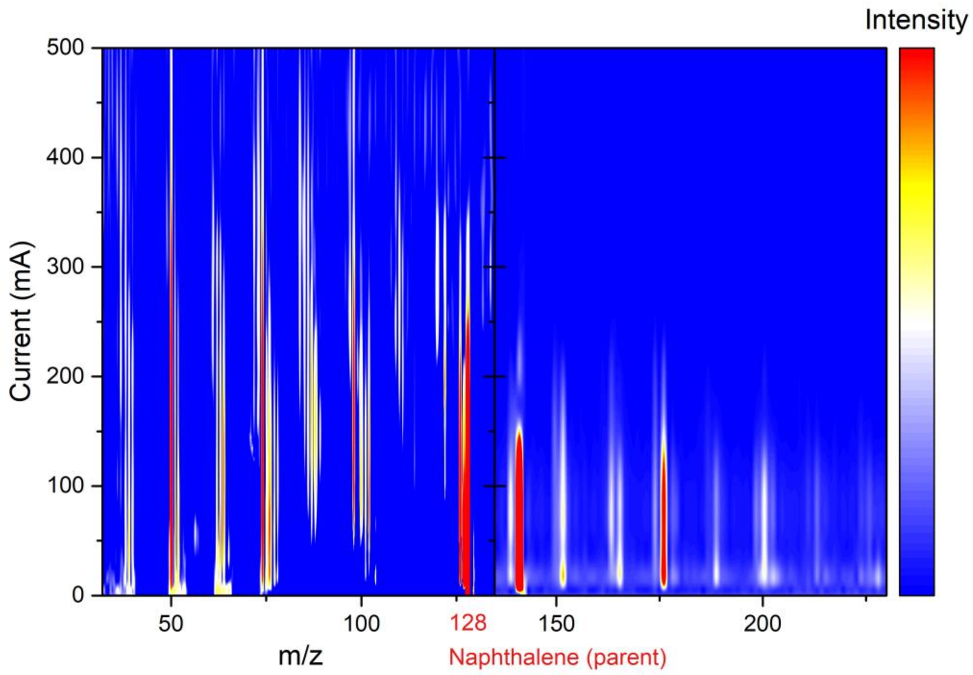

Supplementary Figure 11: Discharge current m/z plot showing the range of currents at which we create fragments and/or products. *When recording the IR-UV ion dip, a current of 50 mA was typical.*

Supplementary References

(1) Bellamy, L. J. *The Infra-Red Spectra of Complex Molecules*; Chapman and Hall: London, 1975.

(2) Constantinidis, P.; Hirsch, F.; Fischer, I.; Dey, A.; Rijs, A. M. Products of the Propargyl Self-Reaction at High Temperatures Investigated by IR/UV Ion Dip Spectroscopy. *J. Phys. Chem. A* **2017**, *121* (1), 181–191.

(3) Nyquist, R. A.; Potts, W. J. Infrared Absorptions Characteristic of the Terminal Acetylenic Group (-C≡C-H). **1960**, *16*, 419–427.

(4) Evans, J. C.; Nyquist, R. A. The Vibrational Spectra and Vibrational Assignments of the Propargyl Halides. *Spectrochim. Acta* **1963**, *19*, 1153–1163.

(5) Wallace, W. E. *Phenylethyne IR Spectrum, NIST Chemistry WebBook, NIST Standard Reference Database Number 69*; P.J. Linstrom and W.G. Mallard, Ed.; National Institute of Standards and Technology: Gaithersburg MD, 2019.

(6) Lemmens, A. K.; Rap, D. B.; Thunnissen, J. M. M.; Mackie, C. J.; Candian, A.; Tielens, A. G. G. M.; Rijs, A. M.; Buma, W. J. Anharmonicity in the Mid-Infrared Spectra of Polycyclic Aromatic Hydrocarbons. Molecular Beam Spectroscopy and Calculations. *Astron. Astrophys.* **2019**, *628* (A130), 1–10.
